# Blood leukocytes recapitulate diabetogenic peptide–MHC-II complexes displayed in the pancreatic islets

**DOI:** 10.1084/jem.20202530

**Published:** 2021-04-06

**Authors:** Anthony N. Vomund, Cheryl F. Lichti, Orion J. Peterson, Ana Maria Arbelaez, Xiaoxiao Wan, Emil R. Unanue

**Affiliations:** 1 Division of Immunobiology, Department of Pathology and Immunology, Washington University School of Medicine, St. Louis, MO; 2 Division of Endocrinology, Department of Pediatrics, Washington University School of Medicine, St. Louis, MO; 3 Bursky Center for Human Immunology and Immunotherapy Programs, Washington University School of Medicine, St. Louis, MO

## Abstract

Assessing the self-peptides presented by susceptible major histocompatibility complex (MHC) molecules is crucial for evaluating the pathogenesis and therapeutics of tissue-specific autoimmune diseases. However, direct examination of such MHC-bound peptides displayed in the target organ remains largely impractical. Here, we demonstrate that the blood leukocytes from the nonobese diabetic (NOD) mice presented peptide epitopes to autoreactive CD4 T cells. These peptides were bound to the autoimmune class II MHC molecule (MHC-II) I-A^g7^ and originated from insulin B-chain and C-peptide. The presentation required a glucose challenge, which stimulated the release of the insulin peptides from the pancreatic islets. The circulating leukocytes, especially the B cells, promptly captured and presented these peptides. Mass spectrometry analysis of the leukocyte MHC-II peptidome revealed a series of β cell–derived peptides, with identical sequences to those previously identified in the islet MHC-II peptidome. Thus, the blood leukocyte peptidome echoes that found in islets and serves to identify immunogenic peptides in an otherwise inaccessible tissue.

## Teaser

Assessing the self-antigens causing type 1 diabetes remains challenging. This study shows that blood leukocytes contain immunogenic peptides with compatible sequences to those presented in the pancreatic islets, representing a feasible strategy for probing a target autoimmune tissue.

## Introduction

Recognition of tissue-specific antigens is critical for initiating T cell responses driving autoimmunity. In type 1 diabetes (T1D), pancreatic islets are targeted by finely programmed autoimmune responses, leading to selective demise of the insulin-producing β cells (Anderson and Bluestone, 2005). The primary genetic susceptibility of this disease is conferred by variants of the MHC-II alleles that bind peptide antigens derived from β cells (Todd et al., 1987; Acha-Orbea and McDevitt, 1987; Miyazaki et al., 1990). These peptide–MHC-II complexes form the substrates for immune recognition by autoreactive CD4 T cells, resulting in their activation. Examining the antigenic entities displayed in the islets has substantially facilitated our understanding of the autoimmune process and provided insights into the development of targeted immunotherapies (Clemente-Casares et al., 2012; Unanue, 2014). Yet probing these tissue-derived antigens is limited by their inaccessibility.

Recent investigations examined how β cells convey their immunological information to the adaptive immune system (Vomund et al., 2015; Wan et al., 2018). Examining the non-obese diabetic (NOD) mouse that spontaneously develops autoimmune diabetes confirmed two sites for presentation of diabetogenic antigens: the peripheral lymphoid system and the islet (Wan et al., 2020). Secondary lymphoid tissues, particularly the pancreatic draining LN, are critical for the priming of the diabetogenic T cells (Höglund et al., 1999; Gagnerault et al., 2002; Levisetti et al., 2004); the local presentation in islets further enhances T cell pathogenicity (Melli et al., 2009; Ferris et al., 2014; Carrero et al., 2017). Peripheral lymphoid tissues are consistently sensitized by antigenic products secreted from the β cells obeying glucose stimulation (Wan et al., 2018). These materials (referred to as secretome) contain catabolized peptide fragments generated in the β cell crinosomes (Wan et al., 2018), a set of lysosomal vesicles that degrades excessive insulin granules to maintain cellular homeostasis (Smith and Farquhar, 1966; Halban and Wollheim, 1980; Orci et al., 1984). We have identified immunogenic epitopes from insulin, a prime antigen required for initiation of diabetes in NOD mice (Nakayama et al., 2005), in the crinosomes and the secretome (Wan et al., 2018).

Diabetes development in the NOD mouse depends on the I-A^g7^ MHC-II molecule (Lund et al., 1990; Singer et al., 1998; Gioia et al., 2019), a structural homologue to the human autoimmune HLA-DQ8 (Corper et al., 2000; Latek et al., 2000; Lee et al., 2001). Both molecules select a similar repertoire of self-peptides (Suri et al., 2005). We performed an unbiased analysis of the immunopeptidome of islets isolated from NOD mice and identified β cell–specific peptides bound to I-A^g7^ (Wan et al., 2020). The major peptides that gave rise to autoreactivity derived from the insulin B-chain (InsB) and the C-peptide (InsC), which were also identified in the MHC-II peptidomes of the pancreatic draining LN and spleen (Wan et al., 2020). The InsB-derived sequences were highly compatible with those that activated HLA-DQ8–restricted CD4 T cells isolated from the peripheral blood or the islets in patients with T1D (Yang et al., 2014; Michels et al., 2017; Babon et al., 2016). These findings highlighted the communication process between the islet and peripheral lymphoid tissues. In this study, we further investigated whether tissue-derived antigenic information can be readily acquired at a site of accessibility. We report that upon β cell degranulation, InsB- and InsC-derived peptides are captured by circulating leukocytes via binding to their MHC-II molecules. These MHC-II–bound peptides are identical to those identified in the islet MHC-II peptidome.

## Results

### Blood leukocytes take up insulin peptides after a glucose challenge

We administered glucose by i.p. injection and 30 min later anesthetized the mice and bled them from the heart or the eye. White blood cells (WBCs) were separated from erythrocytes and then tested for presentation of various diabetogenic epitopes. Presentation was probed by CD4 T cell hybridomas recognizing several I-A^g7^–restricted epitopes. Positive responses were indicated by IL-2 production from the T cell hybridomas when cultured with the blood leukocytes without pulse with exogenous antigens.

**Figure 1.**
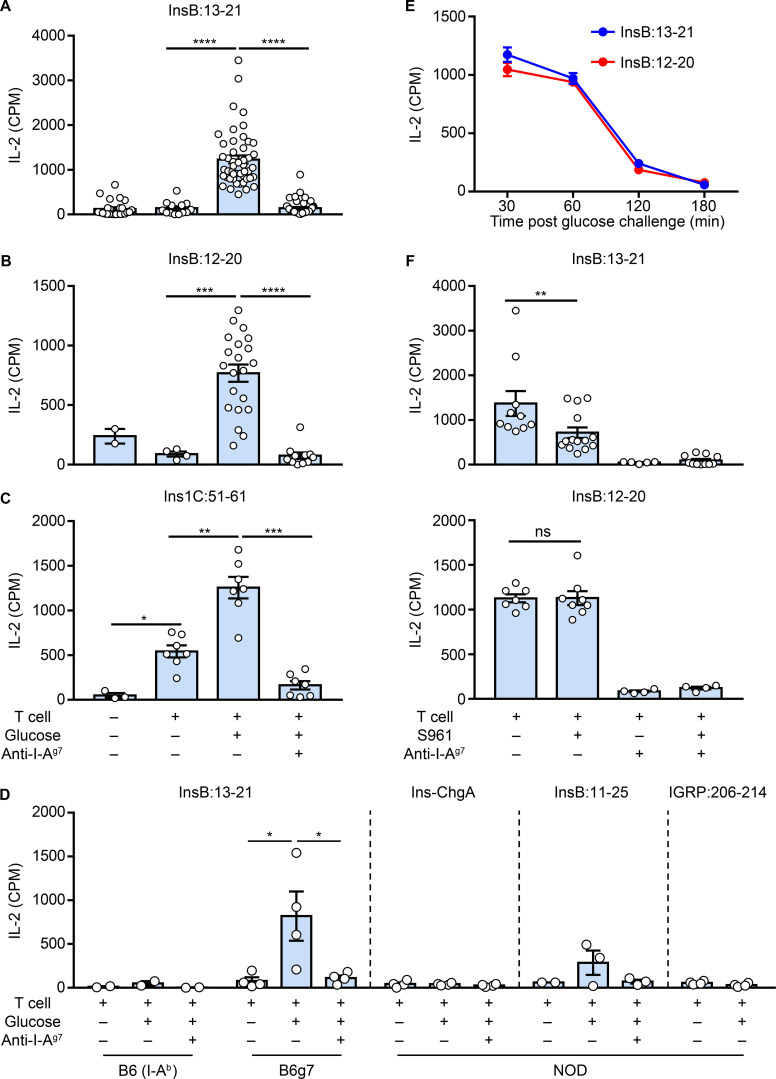
**Spontaneous presentation of immunogenic insulin epitopes by blood leukocytes. (A–C)** Blood leukocytes were obtained from NOD mice with or without a prior glucose challenge and were directly cultured with CD4 T cell hybridomas with defined specificities to InsB:13-21 (A), InsB:12-20 (B), and Ins1C:51-61 (C). IL-2 production from the culture supernatants was measured to indicate the T cell responses. The dependence of MHC-II was determined by adding the anti–I-A^g7^ blocking antibody to the culture. **(D)** Responses of T cell hybridomas with indicated antigen specificities to blood leukocytes obtained from B6, B6g7, or NOD mice. **(E)** Responses of the InsB:13-21– and InsB:12-20–specific CD4 T cells to blood leukocytes obtained from NOD mice at the indicated time points after the glucose challenge. **(F)** Responses of the InsB:13-21– (top) and InsB:12-20– (bottom) specific CD4 T cells to blood leukocytes obtained from NOD mice with or without injection of the insulin receptor blocker S961, followed by glucose challenge. Data (mean ± SEM) summarize results pooled from 2 (D–F), 4 (C), 12 (B), and 28 (A) independent experiments; each point (A–D and F) represents one biological replicate including one to six mice. *, P < 0.05; **, P < 0.01; ***, P < 0.005; ****, P < 0.001; Mann–Whitney test. CPM, counts per minute; Ins-ChgA, insulin–chromogranin A.

Spontaneous presentation of InsB- and InsC-derived epitopes was observed (Fig. 1). We have previously characterized two I-A^g7^–binding registers centered on the 9–23 segment of InsB (InsB:9-23) that generated distinct CD4 T cells (Mohan et al., 2010, 2011; Gioia et al., 2019). The InsB:12-20 register was derived from crinosomes (Wan et al., 2018); this epitope was not generated by canonical processing of insulin but was only presented via extracellular binding of free peptides to MHC-II (Mohan et al., 2010, 2011; Gioia et al., 2019). We have termed it type B presentation. Supporting their pathogenicity, T cells directed to InsB:12-20 escaped thymic selection, underwent peripheral priming, and were among the first to enter islets (Mohan et al., 2013; Wan et al., 2016, 2018; Gioia et al., 2019). A second set of T cells recognized an alternative register, InsB:13-21, which is presented via either intracellular processing of insulin or extracellular peptide binding (Mohan et al., 2010, 2011; Gioia et al., 2019). We refer to these as the conventional type A T cells. Concerning the C-peptide, the C-terminal region of the insulin-1 C-peptide (Ins1C) encompassing residues 51–61 (Ins1C:51-61) was shown to activate autoreactive CD4 T cells (Halbout et al., 2002; Levisetti et al., 2008; Wan et al., 2020). Primary CD4 T cell lines reactive to Ins1C:51-61 were able to transfer diabetes in NOD.Rag1^−/−^ recipients (Wan et al., 2020). In a previous report, we indicated that islets, upon glucose challenge, released not only insulin, as expected, but also a variety of free peptides that resulted from catabolism of insulin and the C-peptide in crinosomes (Wan et al., 2018).

Fig. 1 summarizes all the experiments testing the three epitopes. The blood leukocytes presented InsB:13-21 (Fig. 1 A) and InsB:12-20 (Fig. 1 B) to their specific T cells, but only after a glucose challenge. The responses were low (∼1,000 cpm) but were completely blocked by addition of the anti–I-A^g7^ antibody (Fig. 1, A and B). Therefore, uptake and presentation were specifically mediated by the MHC-II molecule. In contrast to the InsB epitopes, we found a basal level of presentation of the Ins1C:51-61 epitope from WBCs in mice without glucose challenge (Fig. 1 C). Such a response was significantly increased by the glucose challenge (Fig. 1 C). In these experiments, we included female and male NOD mice of different ages (4–16 wk old), and no obvious differences were observed.

Presentation of the InsB-derived peptides was not restricted to the NOD background. WBCs isolated from regular B6 mice (expressing I-A^b^) did not present the InsB:13-21 epitope; by contrast, positive presentation was observed in blood leukocytes from B6 mice harboring the I-A^g7^ haplotype (B6g7; Fig. 1 D). These findings are consistent with our previous observation that crinosomes in B6g7 mice contain immunogenic insulin peptides able to bind to I-A^g7^ (Wan et al., 2018).

We also tested T cells directed to three other diabetes-relevant epitopes, including the InsB:11-25 peptide (Wan et al., 2020), the insulin–chromogranin A fused peptide (recognized by the BDC2.5 T cell; Delong et al., 2016), and the 206–214 peptide from the islet-specific glucose-6-phosphatase catalytic subunit-related protein (IGRP:206-214) presented by the MHC-I molecule K^d^ (recognized by the 8.3 T cell; Lieberman et al., 2003). T cell responses to these peptides were negative after glucose challenge (Fig. 1 D).

Time course experiments indicated that the optimal presentation of InsB:13-21 or InsB:12-20 was observed 30–60 min after the glucose challenge. The responses started to decrease after 60 min and became negative by 180 min (Fig. 1 E). These results implied a very fast interaction between the release of the peptides from islets and the development of the peptide–I-A^g7^ complex.

In vivo uptake of the insulin molecule by APCs was mediated by the insulin receptor; after intracellular processing, the InsB:13-21 epitope was presented (Wan et al., 2018). This epitope was also presented when APCs encountered exogenous peptides (Mohan et al., 2010, 2011; Gioia et al., 2019). To test if these findings apply to blood leukocytes, we administered the specific insulin receptor blocker S961 before glucose challenge and tested presentation. S961 reduced the presentation of InsB:13-21 by ∼50% (Fig. 1 F). Thus, this epitope derived from both internal processing of insulin and direct binding of free peptides. By contrast, S961 administration had no effect in the presentation of InsB:12-20 (Fig. 1 F), an epitope that develops from extracellular peptide binding (Mohan et al., 2010, 2011; Gioia et al., 2019). These findings reinforce the notion that catabolized peptides in the crinosomes are an important antigen source that seeds the periphery upon release from the islets.

### Blood B cells primarily capture and present insulin peptides

**Figure 2.**
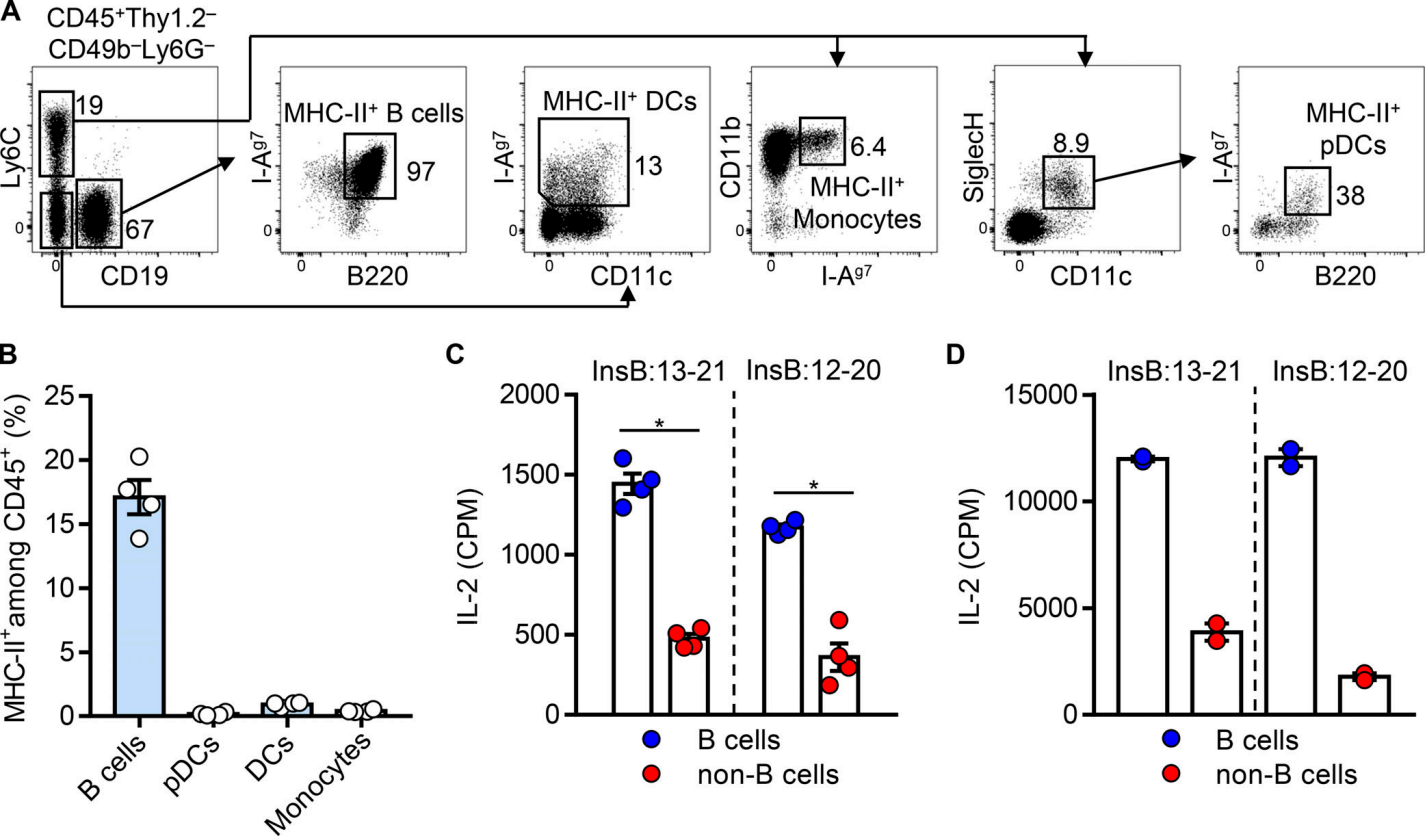
**B cells are the major MHC-II**–**expressing APCs in the blood presenting inulin peptides.** **(A)** Blood leukocytes were obtained from 4-wk-old female NOD mice, and the MHC-II (I-A^g7^)–bearing cells were analyzed by flow cytometry. Data show representative FACS plots depicting the gating strategy for identifying different APCs in the blood, including the MHC-II^+^ B cells, monocytes, pDCs, and conventional DCs. **(B)** Percentage of the MHC-II^+^ B cells, monocytes, pDCs, and conventional DCs, as described in A, among the total CD45^+^ leukocytes in the blood of 4-wk-old female NOD mice. **(C)** Responses of the InsB:13-21– and InsB:12-20–specific CD4 T cells to equal numbers of B cells or non–B cell APCs (regardless of MHC-II expression) isolated from NOD mice given glucose. **(D)** Responses of the InsB:13-21– and InsB:12-20–specific CD4 T cells to equal numbers of B cells or non–B cell APCs isolated from NOD mice (regardless of MHC-II expression) without glucose challenge upon pulse with exogenous InsB:9-23 (10 µM). Data (mean ± SEM) summarize results pooled from two (B–D) independent experiments; each point represents one biological replicate including three or four mice. *, P < 0.05; Mann–Whitney test. CPM, counts per minute.

To identify the MHC-II–bearing APCs in the circulation, we examined WBCs isolated from 4-wk-old female NOD mice by flow cytometry. T cells, natural killer cells, and neutrophils were excluded from the total CD45^+^ leukocytes. The remaining cells (CD45^+^Thy1.2^–^CD49b^–^Ly6G^–^) contained B cells (CD19^+^B220^+^), monocytes (Ly6C^+^CD11b^+^), plasmacytoid dendritic cells (pDCs; Ly6C^+^CD11c^+^SiglecH^+^B220^+^), and conventional DCs (Ly6C^–^CD11c^+^; Fig. 2 A). Among these, the major population was B cells, which ubiquitously expressed I-A^g7^ (Fig. 2 A). By contrast, the monocytes, pDCs, and conventional DCs were limited by cell number, and only a minor portion of these cells expressed I-A^g7^ (Fig. 2 A). Quantification from multiple experiments showed that the MHC-II–bearing B cells constituted ∼20% of the CD45^+^ leukocytes in NOD mice, whereas other APCs had a minimal presence (Fig. 2 B). Thus, B cells are the major APCs in the blood.

We next isolated an equal number of B cells and non–B cell APCs from NOD mice following a glucose challenge and compared their presentation of insulin peptides. Positive responses from presentation of both InsB:13-21 and InsB:12-20 were detected in the B cells, whereas those from the non–B cell compartment were minimal (Fig. 2 C). The circulating B cells highly expressed MHC-II (Fig. 2 A), which may also contribute to the presentation process. This was tested by pulsing a high concentration of exogenous InsB:9-23 in B cells and non–B cell APCs isolated from mice without a glucose challenge. The B cells showed an approximately twofold higher level of presentation to the InsB:13-21– or the InsB:12-20–specific T cells as compared with the non–B cells (Fig. 2 D). Collectively, the insulin peptides released from the islets are primarily captured and presented by the circulating B cells.

### Anti-InsB antibodies block the leukocyte presentation and reduce diabetes incidence

**Figure 3.**
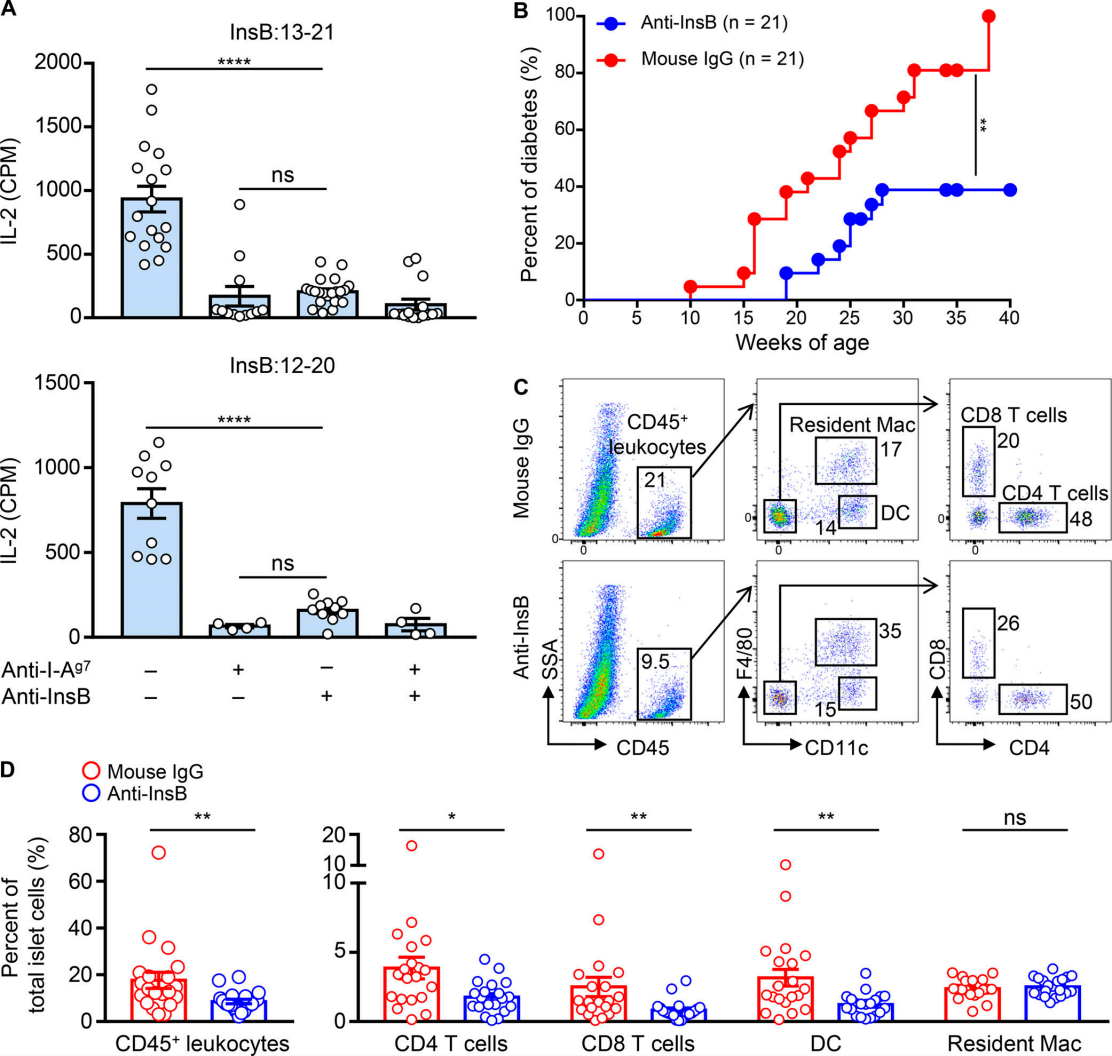
**Specific monoclonal antibodies block the presentation of InsB peptides by WBCs and reduce diabetes penetrance.** **(A)** Responses of the InsB:13-21– and InsB:12-20–specific CD4 T cells to blood leukocytes obtained from NOD mice with or without injection of a mixture of anti–InsB:9-23 and anti–InsB:1-30 antibodies before glucose challenge. The anti–I-A^g7^ antibody was added to the T cell–leukocyte culture as a control. Data (mean ± SEM) summarize results pooled from three independent experiments; each point represents one biological replicate including two to six mice. ****, P < 0.001; Mann–Whitney test. **(B)** Diabetes incidence of female NOD mice administered with the mixture of anti–InsB:9-23 and anti–InsB:1-30 antibodies. The control mice were given polyclonal mouse IgG. Data summarize the percent of diabetes (21 mice per group) from three independent experiments. **, P < 0.005; log-rank test. **(C and D)** Cohorts of female NOD mice were given the anti-InsB antibody mixture or control mouse IgG weekly at 4, 5, and 6 wk of age, and the islets were analyzed for leukocyte infiltration by flow cytometry at 9 wk of age. **(C)** Representative FACS plots showing the gating strategy of the immune cell components in the islets, including the CD45^+^ leukocytes, CD4 and CD8 T cells, DCs, and the resident macrophages (Mac). **(D)** Percentage of the indicated immune cell populations, as depicted in C, among the total islet cells. Data represent results obtained from individual mice (each point) examined in three independent experiments. *, P < 0.01; **, P < 0.005; Mann–Whitney test. CPM, counts per minute; SSA, side scatter A.

We have previously documented two monoclonal antibodies specifically recognizing the InsB:9-23 peptide and the entire InsB (InsB:1-30). These two antibodies did not recognize the conformational insulin molecule and were not cross-reactive (Wan et al., 2018). Considering that both InsB:9-23 and InsB:1-30 were identified as individual sequences in the secretome (Wan et al., 2018) and may therefore provide the InsB:12-20 and InsB:13-21 epitopes, we tested whether administration of these two antibodies may interfere with the presentation. We administered the two antibodies a day before glucose challenge and found a significant blockade of the presentation to the InsB:12-20– and InsB:13-21–specific T cells by the WBCs (Fig. 3 A). The degree of blockade by the anti-InsB antibodies was comparable to that mediated by the anti–I-A^g7^ antibody (Fig. 3 A).

To evaluate whether the blockade of insulin peptide presentation may influence the autoimmune process, we tested the two anti-InsB monoclonal antibodies for their long-term effects in diabetes development. A weekly dose of the antibody mixture was administered to female NOD mice at different ages during the prediabetic stage, and the mice were followed until 40 wk of age. A significant reduction of diabetes incidence was observed in mice recipient of the anti-InsB antibodies relative to those given the control mouse IgG (Fig. 3 B).

We next examined the influence of the antibodies in modulating immune cell infiltration in islets. The antibody mixture was given to female NOD mice at the ages of 4, 5, and 6 wk, and their islets were examined by flow cytometry at the age of 9 wk. We found an ∼50% reduction of the CD45^+^ leukocytes in the islets isolated from mice given the anti-InsB antibodies (Fig. 3 C). Specifically, the infiltration of the CD4 and CD8 T cells as well as the DCs was significantly reduced, and the percentage of the islet resident macrophages was not affected (Fig. 3 D). In sum, specific monoclonal antibodies blocked the capture of insulin peptides released from the islets; such a process may translate into immunoinhibitory effects that dampened the penetrance of diabetes.

### The blood leukocyte MHC-II peptidome includes immunogenic insulin peptides

The MHC-II peptidome of the blood leukocytes in NOD mice following a glucose challenge was examined in two independent analyses that included 20 and 40 NOD mice, respectively. Blood leukocytes were isolated from NOD mice 30 min after glucose challenge. Following cell lysis, the I-A^g7^ molecules were isolated via antibody binding; the peptides bound to them were eluted and examined by liquid chromatography–tandem mass spectrometry (LC-MS/MS). From the two biological samples, 536 peptides were identified, grouped into 186 overlapping sets or families, each having differences in their terminal residues (Table S1, Table S2, and Table S3). In terms of tissue origin, many originated from plasma proteins, including albumin and hemoglobin, while others were from proteins ubiquitously expressed.

The β cell–derived proteins included families centered on the insulin-2 B-chain (Ins2B) and the C-peptide. Of these, the most abundant were derived from the C-terminal (Ins1C:51-61) and the N-terminal (Ins1C:37-56) fragments of Ins1C as well as from Ins2B:9-23 (Table 1, Table S1, Table S2, and Table S3). The InsB and InsC peptide families were independently identified in both experiments (Table 1).

**Table 1.** β cell–derived peptide families identified in the MHC-II peptidome of blood leukocytes

Description	Protein coverage	Sequence	Glucose	No glucose
Ins1C	Ins1C:37–56	PQVEQLELGGSPGDLQTLAL	22, 18	8
Ins2B	Ins2B:9–23	SHLVEALYLVCGERG	10, 11	6
Ins1C	Ins1C:51–61	LQTLALEVARQ	7, 6	0
Insulin-2 C-peptide	Ins2C:37–47	PQVAQLELGGG	2, 2	1
Vitamin D–binding protein	Gc:389–405	SPLLKRQLTSFIEKGQE	1, 1	0
60-kD heat shock protein	Hspd1:164–183	TPEEIAQVATISANGDKDIG	0, 2	0
Zinc transporter 8	ZnT8:313–322	ILSVHVATAA	0, 1	0
Insulin-2 C-peptide	Ins2C:48–58	PGAGDLQTLAL	0, 1	0

MHC-II–bound peptides were isolated from blood leukocytes from NOD mice after glucose challenge and analyzed by LC-MS/MS. The table summarizes all the peptide families derived from pancreatic β cell proteins. The numbers of the individual peptides belonging to each family identified in mice subjected to a glucose challenge (two independent experiments) relative to those without glucose challenge (a single experiment) are shown. The two experiments of the glucose challenge condition used 3 × 10^7^ total blood leukocytes from 20 mice and 7.5 × 10^7^ cells from 40 mice, respectively. The experiment of the no glucose condition used 3 × 10^7^ cells isolated from 19 mice. Columns are as follows: Description, protein name; Protein coverage, gene name and amino acid residues covered; Sequence, amino acid sequence encompassing all peptides identified for a particular family; Glucose, the number of peptides identified in the specified family for the two biological replicates of the glucose challenge experiments; and No glucose, peptides identified in the mice that did not receive glucose.

**Figure 4.**
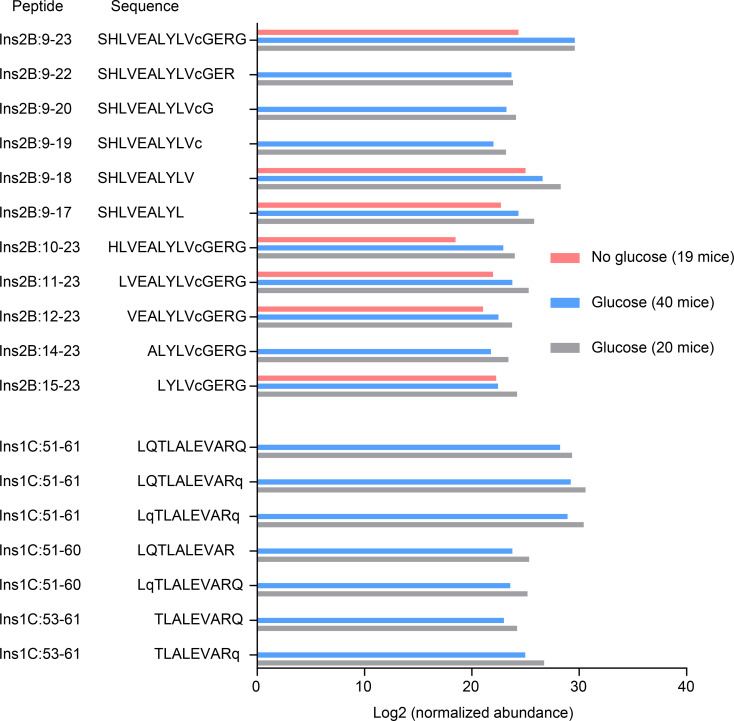
**Relative abundance of insulin peptides isolated from blood leukocytes.** The data show the relative abundance of each individual peptide sequence derived from the Ins2B:9-23 and Ins1C:51-61 families depicted in Table 1. LC-MS/MS data files of the leukocyte MHC-II peptidome were loaded into Skyline for quantification of peptides covering the Ins2B:9-23 and Ins1C:51-61 families, and the resulting peak areas were exported, normalized based on the total ion current, and log2-transformed. The bars represent the normalized log2 abundance of the indicated peptides. The results of the no glucose condition represent a single experiment analyzing 3 × 10^7^ blood leukocytes from 19 NOD mice without glucose challenge. The data of the glucose challenge condition are from two independent experiments including 20 (3 × 10^7^ cells) and 40 (7.5 × 10^7^ cells) NOD mice after glucose injection, respectively. In peptide sequences, the lowercase c denotes the oxidation of cysteine to cysteic acid, and the lowercase q denotes the deamidation of glutamine.

At the peptide level, we identified 12 sequences covering Ins2B:9-23 (Fig. 4), including Ins2B:9-23, Ins2B:11-23, and Ins2B:12-23. All these three peptides were shown to activate the InsB:12-20– or InsB:13-21–specific T cells (Wan et al., 2018, 2020). For Ins1C:51-61, seven peptide sequences were identified (Fig. 4). Most sequences varied only by deamidation, a posttranslational modification that converts glutamine (Q) to glutamic acid (E), at Q52 and/or Q61; singly deamidated (Q61) and doubly deamidated forms of the peptide each comprised approximately one third of the Ins1C:51-61 pool (Fig. 4). Of note, because I-A^g7^ favors binding of peptides having acidic residues at the C terminus (Corper et al., 2000; Latek et al., 2000; Lee et al., 2001; Suri et al., 2005), the deamidation change at Q61 enhanced the binding of the Ins1C:51-61 peptide to I-A^g7^ (Wan et al., 2020). As a result, the deamidated Ins1C:51-61 (LQTLALEVARE) elicited a stronger T cell response than the native version (LQTLALEVARQ; Wan et al., 2020).

MHC-II–bound peptides were also isolated from WBCs of 19 NOD mice without challenging with glucose (Table S4). In general, fewer peptides belonging to each β cell–derived family were identified in this sample (Table 1 and Table S4). Moreover, the relative abundances (normalized to total ion current; see Materials and methods) of the identified insulin peptides were lower than those after glucose challenge (Fig. 4). This is consistent with the results of antigen presentation experiments.

Importantly, the peptides identified in the WBC MHC-II peptidome overlapped with our previously published islet and spleen peptidomes (Wan et al., 2020), with 43/186 families also identified in islets and 57/186 in the spleen (Table S1). All the β cell–derived families identified in the WBC peptidome (Table 1) were found in that of the islet and spleen. We did not find unique β cell peptides in the WBC peptidome. In particular, many of the Ins2B- and Ins1C-derived peptides had identical sequences with those presented in the islets. Identification of the Ins2B peptides with oxidation of cysteine and the InsCs with deamidation (Fig. 4) were consistent with our findings in islets and spleen peptidome (Wan et al., 2020). Other common β cell epitopes, including those from secretogranin and hybrid insulin peptides, were not found in the WBC peptidome. These families showed a lower abundance than insulin peptides in the islets (Wan et al., 2020) and may therefore remain below the limit of detection by MS in the WBCs.

## Discussion

This study shows that circulating leukocytes contain immunogenic peptide–MHC-II complexes shortly after a glucose challenge. We demonstrate spontaneous presentation of peptide epitopes from proinsulin by MHC-II^+^ blood leukocytes, particularly the B cells. The documented presentation is specific and pathologically relevant. The administration of antibodies reactive to InsB peptides blocks the recognition of InsB-derived epitopes and dampens diabetes development. Moreover, the MHC-II peptidome of the WBCs contains the major series of β cell–derived peptides identified in the islets peptidome, including identical sequences of immunogenic InsB- and InsC-derived peptides. Collectively, these findings demonstrate that peripheral blood is an accessible site for probing pancreatic islets. In the context of T1D, this strategy may facilitate the evaluation of disease diagnosis and pathogenesis.

Examining the WBCs provides a window for assessing the immunogenic peptides of the target organ. In support of this concept, elegant studies have shown that blood B cells from patients with multiple sclerosis presented self-peptides to brain-homing CD4 T cells (Jelcic et al., 2018). Such presentation led to autologous T cell proliferation and depended on the expression of the HLA-DR15 haplotype (Jelcic et al., 2018; Mohme et al., 2013; Wang et al., 2020), a major genetic risk factor of multiple sclerosis. DCs carrying antigens from peripheral tissues were shown to enter the circulation, from where they migrated to the bone marrow and mediated activation of resident memory T cells during antigen-specific interactions (Cavanagh et al., 2005). A recent study identified unexpected T cell–monocyte doublets in the blood of human individuals; intriguingly, the frequency and the functional signature of these cell complexes reflected the immune history of different individuals with infection or immunization (Burel et al., 2019). Future studies that emphasize the features of the immunopeptidome in the blood during immunological challenges should be highly informative.

The main APCs spontaneously presenting insulin peptides in mouse blood are B cells, owing to their high number and expression of MHC-II. We have documented that the MHC-II peptidome of B cells purified from the spleen of NOD mice contained InsB- and InsC-derived peptides (Wan et al., 2020). These sequences are compatible with those in the WBC peptidome described in this study. These results suggest that blood B cells may ferry antigens into secondary lymphoid tissues in a cell-associated manner. Peripheral B cells may also directly acquire antigens via uptake of insulin peptides from the blood. This was supported by the finding that fluorescently labeled InsB:9-23 peptide rapidly entered the spleen and bound to I-A^g7+^ B cells shortly after intravenous injection (Wan et al., 2018). Dissecting the antigen sources may facilitate a better understanding of the indispensable role of B cells in driving diabetes development in NOD mice (Serreze et al., 1998). The importance of antigen presentation by B cells was highlighted by previous studies showing that genetic ablation of I-A^g7^ in B cells largely abolished diabetes onset (Noorchashm et al., 1999). We and others also demonstrated that presentation of diabetogenic antigens by peripheral B cells assisted T cell autoreactivity (Greeley et al., 2001; Mariño et al., 2012; Hu et al., 2007; Wan et al., 2016; Kendall et al., 2013; Henry et al., 2012; Hulbert et al., 2001). It is tempting to speculate that the capture of islet-derived peptides by the circulating B cells is an integral component of B cell–mediated pathogenicity.

We documented that administration of anti-InsB monoclonal antibodies completely blocked the presentation of the InsB:13-21 and InsB:12-20 epitopes by WBCs. The two monoclonal antibodies were generated by immunizing with InsB:9-23 or InsB:1-30, respectively; both peptides were identified as independent sequences released from the islets (Wan et al., 2018). InsB:9-23 is a well-established pathogenic peptide required for initiation of the autoimmune process in NOD mice (Nakayama et al., 2005, 2007); in humans with T1D, CD4 T cells reactive to this peptide have been isolated (Michels et al., 2017). The anti–InsB:9-23 antibody identified crinosomes in the islets, a compartment rich in short insulin peptides (Wan et al., 2018). The InsB:1-30 peptide derived from denatured insulin and was mainly found in the regular insulin granules along with conformational insulin molecules (Wan et al., 2018). Presentation of this long peptide did not require internal processing and was able to activate the type B T cells (Wan et al., 2018). The blockade of the leukocyte presentation indicates that InsB:9-23 and InsB:1-30 represent the major native peptides harboring the defined immunogenic epitopes, which seed the periphery.

The inhibition of diabetes progression by the antibody mixture may result from clearance of the InsB peptides from the circulation. Considering the fast turnover of the peptide–MHC-II complexes observed in this study, we are testing different protocols involving persistent administration of the antibodies, which may result in a better control. Moreover, this finding raises the possibility that combining antibodies directed to other immunogenic peptides, such as the C-terminal segments of Ins1C, may improve the therapeutic effects. Whether targeting MHC-I–dependent peptides may exert similar effects remains to be determined.

## Materials and methods

### Data availability

The MS data are available via ProteomeXchange with the dataset identifier PXD024400.

### Mice

NOD/ShiLtJ (NOD), C57BL/6J (B6), and B6.NOD-(D17Mit21-D17Mit10)/LtJ (B6g7) mice were originally obtained from The Jackson Laboratory. Mice were bred and maintained under specific pathogen–free conditions in our facilities. All experiments were approved by the division of Comparative Medicine of Washington University School of Medicine in St. Louis (accreditation number A3381-01).

### Isolation of blood leukocytes

Mice were fasted overnight, and then administered i.p. 4 g glucose/kg of body weight in saline solution. After a period of time, usually 30 min in most experiments, the mice were anesthetized, and blood was drawn from the heart or from the eye into a heparinized vessel. To isolate the WBCs, the blood was mixed with an equal volume of DMEM containing 10% fetal bovine serum. The diluted blood sample was added to a 15-ml conical tube and underlaid with an equal volume of Histopaque 1077 (Sigma-Aldrich; #H8889). Samples were spun for 35 min at 400 *g*. The WBCs were isolated from the interface and washed twice with PBS.

### Treatment with S961 and anti-InsB antibodies

For S961 (Phoenix Pharmaceuticals) treatment, the animals were injected with 10 nmol in water i.p. 30 min before the glucose injection. For testing the anti-InsB antibodies in WBC presentation, mice were injected i.p. with 500 µg each of the two monoclonal antibodies, anti-InsB:9-23 (clone AIP) and anti-InsB:1-30 (clone 6F3.B8), or 1 mg of polyclonal mouse IgG (BioXcell), and 18 h later, the mice were challenged with glucose. For treating diabetes, the antibody mixture (250 µg per antibody per injection) or mouse IgG (500 µg per injection) was administered i.p. weekly in female NOD mice at the age of 4, 5, 6, 9, 10, 11, 14, 15, and 16 wk. The mice were monitored for diabetes incidence weekly until the age of 40 wk. Mice with two consecutive readings of blood glucose >250 mg/dl were considered diabetic. For testing islet infiltration, the antibody mixture (250 µg per antibody per injection) or mouse IgG (500 µg per injection) was given i.p. weekly in female NOD mice at the ages of 4, 5, and 6 wk. The mice were sacrificed at the age of 9 wk, and the islets were examined by flow cytometry.

### Enrichment of B cells and non–B cell APC populations from blood

WBCs were harvested as described and were first depleted of T cells by anti-CD3 magnetic beads (Miltenyi Biotec). B cells were then positively selected for using anti-CD19 magnetic beads (Miltenyi Biotec). The unbound cells containing non–B cell APCs were subjected to a second round of CD19 selection to remove the residual B cells. Flow cytometry analysis showed high (>95%) purity of the B cells and the non–B cell APCs (monocytes, pDCs, and DCs).

### Antigen presentation assays

Isolated WBCs (10^5^ per well in 96-well plates) were cultured overnight with a variety of different T cell hybridomas (5 × 10^4^ per well): IIT-3 (InsB:13-21), 9B9 (InsB:12-20), 3526–51 (Ins1C:51-61), BDC2.5 (insulin–chromogranin A), clone 58 (InsB:11-25), and 8.3 (IGRP 206–214). The T cell responses were probed by IL-2 production measured by culturing supernatants in the presence of the IL-2–dependent cell line CTLL-2. The proliferation of CTLL-2 cells was measured by the incorporation of ^3^H-thymidine.

### Isolation of pancreatic islets

Upon exposure of the pancreas, the duodenum ampulla was clamped under a dissection microscope. Via the common bile duct, the pancreas was inflated with 5 ml calcium-free Hank’s balanced salt solution containing type XI collagenase (0.4 mg/ml; Sigma-Aldrich). The pancreas was then excised and digested for 15 min at 37°C. The digestion mixture was briefly shaken for 90 s and passed through a 70-µm cell strainer. The islet-containing fraction retained by the strainer was flushed into a Petri dish for handpicking. The handpicked islets were dispersed into single-cell suspensions by incubating in the enzymatic-free cell dissociation solution (Sigma-Aldrich) for 3 min at 37°C. Islet isolation was performed in individual mice, which generated ∼100 islets per mouse. All the islets isolated from the same mouse were pooled as one biological sample for subsequent examination.

### Flow cytometry analysis

Cell suspensions were prepared from blood leukocytes or dispersed islet cells. The cells were incubated in a PBS-based buffer containing 0.5% BSA and 2.4G2 antibody (10 µg/ml) for 20 min on ice to block Fc receptors. The following antibodies purchased from Biolegend were used to stain the cells for 25 min on ice in the same buffer: Brilliant violet–anti-CD45 (30-F11), PE–anti-Thy1.2 (30-H12), PE–anti-CD49b (HMα2), PE–anti-Ly6G (1A8), FITC–anti-B220 (RA3-6B2), PerCP-Cy5.5–anti-Ly6C (HK1.4), APC-Cy7–anti-CD11c (N418), PE-Cy7–anti-CD11b (M1/70), APC–anti-SiglecH (551), FITC–anti-CD4 (RM4-5), Pacific Blue–anti-CD8a (53–6.7), and APC–anti-F4/80 (BM8). The anti–I-A^g7^ antibody (clone AG2.42.7) was generated in the laboratory and conjugated to Pacific Blue using a labeling kit (Invitrogen; P30012). The cells were washed twice and acquired using a FACSCanto II (BD Biosciences), and data were analyzed using FlowJo software (Tree Star).

### Isolation of MHC-II peptidome

We followed our original procedure (Wan et al., 2020), with modifications. The WBCs were suspended in lysis buffer (40 mM N-octanoyl-N-methylglucamine, 40 mM N-nonanoyl-N-methylglucamine, 1 mM phenylmethylsulfonyl fluoride, 0.2 mM iodoacetamide, 20 µg ml^−1^ leupeptin, and Roche cOmplete Mini Protease cocktail in PBS) and rocked for 1 h at 4°C. The cell lysate was spun in a centrifuge at 20,000 *g* for 30 min at 4°C. To eliminate nonspecific binding of peptides, the supernatant was first incubated with polyclonal mouse IgG (1.5 mg antibody per sample; Bio X Cell) bound to sepharose 4B at 4°C for 30 min. The unbound material containing MHC-II–peptide complexes was collected and was then added to a tube containing PBS-washed sepharose conjugated to the anti–I-A^g7^ antibody (1.5 mg per sample; AG2.42.7) and incubated at 4°C overnight. The I-A^g7^–sepharose was applied to a column and washed four times as follows: 10 ml 150 mM NaCl and 20 mM Tris, pH 7.4; 10 ml 400 mM NaCl and 20 mM Tris, pH 7.4; 10 ml 150 mM NaCl and 20 mM Tris, pH 7.4; and 10 ml 20 mM Tris, pH 8.0. Peptides were eluted with 10% acetic acid and dried with a SpeedVac. Eluted peptides were passed over detergent removal spin columns (Pierce) to remove traces of remaining detergent and were cleaned using C18 Spin Columns from Thermo Fisher Scientific (Pierce).

### MS analysis and data analysis

A Dionex UltiMate 1000 system (Thermo Fisher Scientific) was coupled to an Orbitrap Fusion Lumos (Thermo Fisher Scientific) through an Easy-Spray ion source (Thermo Fisher Scientific). Peptide samples were dissolved in 2% acetonitrile/0.1% formic acid (20 µl), loaded (19 µl, 15 µl/min, 3 min) onto a trap column (100 µm × 2 cm, 5 µm Acclaim PepMap 100 C18, 50°C), eluted (0.200 µl/min) onto an Easy-Spray PepMap RSLC C18 column (2 µm, 50 cm × 75 µm inner diameter, 50°C; Thermo Fisher Scientific), and separated with the following gradient, all percentages of buffer B (0.1% formic acid in acetonitrile): 0–110 min, 2–22%; 110–120 min, 22–35%; 120–130 min, 35–95%; 130–150 min, isocratic at 95%; 151–153 min, 95–2%; 153–171 min, isocratic at 2%. Spray voltage was 1,900 V, ion transfer tube temperature was 275°C, and radio frequency lens was 30%. MS scans were acquired in profile mode and MS/MS scans in centroid mode, for ions with charge states 2–5, with a cycle time of 3 s. MS spectra were recorded from 375 to 1,500 daltons at 120,000 resolution (at mass to charge 200), and higher energy collision-induced dissociation MS/MS was triggered above a threshold of 2.0e4, with quadrupole isolation (0.7 daltons) at 30,000 resolution, and collision energy of 30%. Dynamic exclusion was used (±5 parts per million, 60 s), and monoisotopic precursor selection was on.

### MS data analysis and quantification

Data files were uploaded to PEAKS X+ (version 10.5; Bioinformatics Solutions) for processing, de novo sequencing, and database searching (Tran et al., 2019). Resulting sequences were searched against the UniProt Mouse database (downloaded January 12, 2019; 17,000 entries) appended with the Common Repository for Adventitious Proteins contaminant database, with mass error tolerances of 10 parts per million and 0.01 daltons for parent and fragment, respectively, no enzyme specificity and methionine oxidation, deamidation of glutamine and asparagine, and oxidation of cysteine to cysteic acid as variable modifications. False discovery rate estimation was enabled, and filters were adjusted to give a 1% false discovery rate at the peptide level unless otherwise noted.

To measure peptide abundances, data files were imported into Skyline (64 bit, version 20.1.0.155 [a0e7323e3], MacCoss Laboratory, University of Washington; Schilling et al., 2012). The resulting abundances were exported, normalized to the total ion current, log2 transformed, and plotted in SigmaPlot 13.0 (build 13.0.0.83; Systat Software).

### Statistics

The Mann–Whitney test was used to determine the statistical differences between two experimental groups with unpaired biological replicates. The log-rank test was used to determine the significant difference in diabetes incidence.

### Online supplemental material

Table S1 summarizes all the peptide families identified in the blood leukocytes from NOD mice with or without challenge with glucose. Table S2 summarizes all the individual peptides identified in the MHC-II peptidome of the blood leukocytes isolated from 40 NOD mice after glucose challenge. Table S3 summarizes all the individual peptides identified in the MHC-II peptidome of the blood leukocytes isolated from 20 NOD mice after glucose challenge. Table S4 summarizes all the individual peptides identified in the MHC-II peptidome of the blood leukocytes isolated from 10 NOD mice without glucose challenge.

## Supplementary Material

Table S1 is a compiled list of peptide families identified in WBCs isolated from NOD mice, both with (NOD_40 mice and NOD_20 mice) and without (control_19 mice) glucose challenge. The number in each column indicates the number of peptides identified.

Table S2 is a list of MHC-II peptides identified by LC-MS/MS from PBMCs isolated from NOD mice after glucose challenge (40 mice).

Table S3 is a list of MHC-II peptides identified by LC-MS/MS from PBMCs isolated from NOD mice after glucose challenge (20 mice).

Table S4 is a list of MHC-II peptides identified by LC-MS/MS from PBMCs isolated from NOD mice without glucose challenge (control).
